# Associations of Humidity and Temperature With Cataracts Among Older Adults in China

**DOI:** 10.3389/fpubh.2022.872030

**Published:** 2022-03-31

**Authors:** Xiaoyang Lv, Xiangyang Gao, Kejia Hu, Yao Yao, Yi Zeng, Huashuai Chen

**Affiliations:** ^1^School of Public Health, Cheeloo College of Medicine, Shandong University, Jinan, China; ^2^The Second Medical Center and National Clinical Research Center for Geriatric Diseases, Health Management Institute, Chinese PLA General Hospital, Beijing, China; ^3^School of Public Health, Institute of Big Data in Health Science, Zhejiang University, Hangzhou, China; ^4^China Center for Health Development Studies, Peking University, Beijing, China; ^5^Center for Healthy Aging and Development Studies, National School of Development, Peking University, Beijing, China; ^6^Business School of Xiangtan University, Xiangtan, China

**Keywords:** relative humidity, temperature, extreme temperature, cataracts, older adults, China

## Abstract

**Background:**

The burden of cataracts was substantial in the current aging world. However, few epidemiological studies have examined the associations between climate and weather conditions and cataract in older populations. We aimed to investigate the associations of air relative humidity and temperature with cataracts in older adults in China.

**Methods:**

We used the cohort data from 2002, 2005, 2008, 2011, 2014, and 2018 waves of the Chinese Longitudinal Healthy Longevity Survey (CLHLS). A total of 62,595 Chinese older adults aged between 65 and 105 years were included in the analyses. City-level annual average air humidity and temperature during 2001 and 2017 (before the survey year) was used to measure population exposure. A cataract was self-reports based on the medical record or the doctor's diagnosis and 8,071 older adults had cataract. Covariates included socio-demographic, health status, lifestyles, and chronic conditions. We adopted the Generalized estimation equation (GEE) model to analyze the associations of relative humidity and temperature with cataracts.

**Results:**

We found that the average relative humidity (OR: 0.99; 95% CI: 0.98–0.99) in the past year was inversely associated with cataract likelihoods in older adults and a positive association between temperature (OR: 1.04; 95%CI: 1.03,1.05) in the past year and cataract likelihoods in older adults. The associations were robust in stratified analyses by sex, urban/rural residence, and education level. Furthermore, we found a nonlinear J-shaped relationship between temperature and cataract prevalence.

**Conclusion:**

Our findings provide the evidence that higher temperature and low relative humidity may be associated with cataracts in older adults.

## Introduction

Cataract, defined as any opacity of the crystalline lens in the eye that affects clear vision, is a common condition in later life (1, 2). Cataracts are the second leading cause of visual impairment and the first cause of blindness worldwide (3). In addition to the reduced vision-related quality of life, people with cataracts are at a higher risk of comorbidity and mortality (4). The only effective treatment for cataracts is cataract surgery, which is still very expensive in developing countries. The Chinese population is experiencing rapid aging, which will increase the burden of cataracts and cataract blindness. As of 2050, cataract cases in China aged 45–89 are predicted to more than double to 240.83 million, with a high prevalence of one-third (33.34%) (5). To identify the modifiable risk factors for cataracts in an aging society such as China is imperative for disease prevention and control.

Environmental factors-induced adverse health outcomes were well-documented (6). A large number of studies have observed both short-term and long-term effects of weather conditions (e.g., temperature, humidity) on human health, such as cardiopulmonary diseases, urinary system diseases and rheumatoid arthritis (7, 8). Studies have also suggested a relationship between both humidity and temperature and eye diseases (9). Zhong et al. found that relative humidity was negatively related to allergic conjunctivitis and there was a 5.8% reduction in allergic conjunctivitis occurrence for every 10% increase in relative humidity (10). A cross-sectional study of South Korean population revealed a negative association between dry eye disease and relative humidity levels (11). In another study, a dry eye disease diagnosis was negatively associated with humidity levels, positively with temperature and sunshine duration (12). A recent study also showed that the relative humidity was negatively, while the temperature was positively, associated with dry eye disease (13). However, the effects of long-term exposure to cold or warm temperatures and high or low humidity levels on cataracts are still poorly understood.

In light of the increasing occurrence of extreme climates and extreme temperatures, it is essential to examine their impacts on health, especially that of aging individuals, which will be directly related to future healthy aging and the creation of age-friendly environments. However, epidemiological studies on cataracts in older adults are rare. This study examined the associations of humidity and temperature with cataracts among a nationally representative sample of older adults in China.

## Method

### Study Population

Data used in this study were derived from the Chinese Longitudinal Healthy Longevity Survey (CLHLS) waves of 2002, 2005, 2008, 2011, 2014, and 2018. The CLHLS applied a multistage, stratified cluster sampling design in 23 out of 31 provinces in China. The goal of CLHLS was to understand better the determinants of healthy longevity in Chinese older adults. Between 1998 and 2018, the CLHLS was conducted in half of the counties and cities (randomly selected) in 23 out of 31 provinces in China. Details of the CLHLS have been described (14). Informed consent was obtained from all participants and/or their relatives, and the Ethics Committee of Peking University approved the study (IRB00001052-13074).

**Figure 1:**
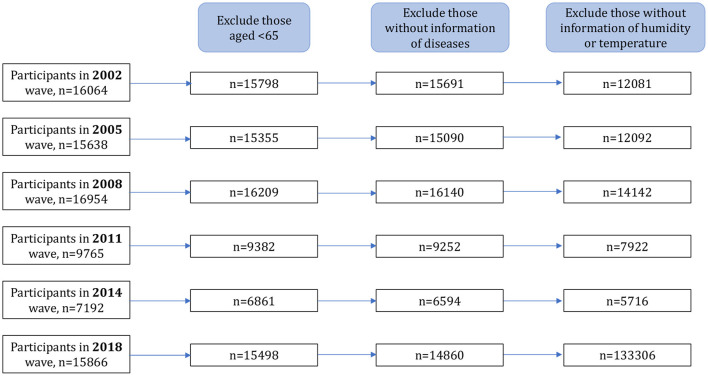
Flow chart of the study.

A total of 62,595 older adults were included in the final analyses by combining all six waves of CLHLS data between 2002 and 2018. Data cleaning as well as inclusion and exclusion criteria are illustrated in the flow chart (Figure 1).

### Assessment in Residential Air Temperature and Relative Humidity

City-level hourly data in 2002, 2005, 2008, 2011, 2014, and 2018 of air relative humidity (%) and temperature (°C) are derived from the China Meteorological Data Network (http://data.cma.cn/). Hourly data was annually or seasonally averaged to measure residential temperature and relative humidity exposure for each participant in CHLHS, matching by the administrative codes of the cities where the CLHLS samples are resident (non-publicly available information). The temperature and humidity exposures adopted in this study are (1) Average relative humidity over the past year and (2) Average temperature in the past year. To reduce the seasonal bias, we also used (3) Average temperature in the cold months and (4) Average temperature in the warm months to measure the participants' temperature exposure. Similar with the previous publications (15), the warm months were defined as the period between May and October, and the average temperature over these 6 months was defined as the average temperature during these months. Cold months refer to January through April and November through December, and the average temperature of these 6 months is equal to the average temperature of those 6 months. The extreme heat was represented by (5) Average of daily maximum temperature, while the extreme cold was represented by (6) Average of daily minimum temperature.

Referring to the previous publications (16, 17), and trying to make the sample numbers of the five grades similar as well as the numerical critical point is rounded, the average relative humidity in the past year was classified into five categories: very low (<60), low (60–69.99), middle (70–74.99), high (75–79.99), very high (≥80). The average temperature in the past year data has been divided into five grades: very low (<5, low: 5–7.99), middle (8–9.99), high (10–12.99), very high (≥13). The average temperature in the warm months of past year data was classified into five grades as classification variables: very low (<21), low (21–22.99), middle (23–24.49), high (24.5–25.99), very high (≥26).

### Cataract

Cataract information was self-reports based on the medical record or the doctor's diagnosis for each participant.

### Covariates

To minimize the effect of potential confounders, we used recent literature in 10 years in PubMed to identify the variables as covariates including common predictors of cataract (1, 2, 18, 19). These variables included age, sex (male or female), residence (urban or rural), geographic region (east provinces or central and western provinces), education (illiterate, having fewer than 6 years of education, or having more than seven years of education), marital status (married or single, which includes divorced, widowed, or never married), and economic status [Log of per capita income = ln(per capita household income+1). Per capita household income (CNY)” represents the total household income in the past 12 months divided by the number of family members]. The definition of disability is any limitation in any activity of daily living, such as bathing, dressing, using the bathroom, indoor transferring, continence, or eating. Cognitive functioning was evaluated using the Chinese version of the Mini Mental State Examination (MMSE), which is one of the most commonly used tools for assessing cognitive health in older adults and documenting cognitive changes as they occur (20, 21).

### Statistical Analyses

This study explored the effect of air relative humidity and temperature on the prevalence of cataracts. Baseline characteristics of participants were summarized based on the presence or absence of cataracts. We presented data as means and standard deviations (SD) for continuous variables and as frequencies and percentages for categorical variables. Associations of relative humidity and temperature with cataracts were analyzed by generalized estimation equation (GEE). We chose GEE because using the observed correlational structure of the data, we can obtain efficient and unbiased regression parameters (22). We used the logit link function and reported the Odds Ratios (OR), and 95% confidence intervals (CIs) obtained from the model estimated robust standard errors. We used an exchangeable correlation structure to account for subject-level repeated measures. Models have been adjusted for potential confounders, including demographic, health, and psychological factors. All statistical analyses were performed using statistical software Stata 14.1.

## Results

### Description of the Study Sample

A total of 62,595 observations were included in this study. The mean age of the participants was 85.78 ± 11.27 years; the gender composition was generally balanced (44.4% men). The prevalence of cataracts was 12.89%. Nearly half (47.6%) of the older adults lived in urban areas, and nearly half (48.6%) lived in east China. More than half (55.6%) of the participants were illiterate, and about 36.2% of them were married. About 25.4% of the participants have disability in activities of daily living, and 20.9% of them had cognitive impairment (Table 1).

**Table 1:** Characteristics of the study samples, CLHLS 2002–2018.

	**All samples**	**No cataracts**	**Having cataracts**	***P*-values of difference**
	***N* = 62,595**	***N* = 54,524**	***N* = 8,071**	
Having cataracts, *n* (%)	8071(12.9)	–	–	–
Average relative humidity in the past year, mean(SD)	71.77(8.01)	71.90(7.89)	70.91(8.76)	<0.001
Grades: Very low: <60, *n*(%)	6510(10.4)	5345(9.8)	1165(14.4)	<0.001
Low: 60–69.99, *n* (%)	16455(26.3)	14453(26.5)	2002(24.8)	0.001
Middle: 70–74.99, *n* (%)	14048(22.4)	12291(22.5)	1757(21.8)	0.120
High: 75–79.99, *n* (%)	16853(26.9)	14810(27.2)	2043(25.3)	0.001
Very high: ≥80, *n* (%)	8729(13.9)	7625(14.0)	1104(13.7)	0.459
Average temperature in the past year, mean (SD)	16.30(3.92)	16.30(3.88)	16.33(4.21)	0.584
Grades: Very low: <12°C, *n* (%)	6145(9.8)	5198(9.5)	947(11.7)	<0.001
Low: 12–14.99°C, *n* (%)	12503(20.0)	10967(20.1)	1536(19.0)	0.023
Middle: 15–16.99°C, *n* (%)	16482(26.3)	14657(26.9)	1825(22.6)	<0.001
High: 17–19.99°C, *n* (%)	18212(29.1)	15862(29.1)	2350(29.1)	0.964
Very high: ≥20°C, *n* (%)	9253(14.8)	7840(14.4)	1413(17.5)	<0.001
Average temperature in the cold months of past year, mean(SD)	8.86(5.57)	8.86(5.50)	8.84(5.99)	0.715
Grades: Very low: <5°C, *n* (%)	11053(17.7)	9350(17.1)	1703(21.1)	<0.001
Low: 5–7.99°C, *n* (%)	13800(22.0)	12433(22.8)	1367(16.9)	<0.001
Middle: 8–9.99°C, *n* (%)	11568(18.5)	10050(18.4)	1518(18.8)	0.417
High: 10–12.99°C, *n* (%)	14865(23.7)	12973(23.8)	1892(23.4)	0.489
Very high: ≥13°C, *n* (%)	11309(18.1)	9718(17.8)	1591(19.7)	<0.001
Average temperature in the warm months of past year, mean (SD)	23.64(2.42)	23.63(2.39)	23.70(2.57)	0.011
Grades: Very low: <21°C, *n* (%)	6756(10.8)	5750(10.5)	1006(12.5)	<0.001
Low: 21–22.99°C, *n* (%)	13919(22.2)	12371(22.7)	1548(19.2)	<0.001
Middle: 23–24.49°C, *n* (%)	19757(31.6)	17376(31.9)	2381(29.5)	<0.001
High: 24.5–25.99°C, *n* (%)	12832(20.5)	11084(20.3)	1748(21.7)	0.006
Very high: ≥26°C, *n* (%)	9331(14.9)	7943(14.6)	1388(17.2)	<0.001
Average of daily maximum temperature in the past year, mean (SD)	21.08(3.62)	21.08(3.58)	21.11(3.88)	0.495
Average of daily minimum temperature in the past year, mean (SD)	12.63(4.54)	12.63(4.49)	12.62(4.87)	0.801
**Covariates**				
Living in urban area, *n* (%)	29809(47.6)	24872(45.6)	4937(61.2)	<0.001
East China, *n* (%)	30425(48.6)	25956(47.6)	4469(55.4)	<0.001
Male, *n* (%)	27787(44.4)	24808(45.5)	2979(36.9)	<0.001
Age, mean (SD)	85.78(11.27)	85.40(11.33)	88.38(10.53)	<0.001
Age group: 65–79, *n* (%)	20076(32.1)	18206(33.4)	1870(23.2)	<0.001
80–89, *n* (%)	16376(26.2)	14239(26.1)	2137(26.5)	0.489
90–99, *n* (%)	15736(25.1)	13419(24.6)	2317(28.7)	<0.001
100–105, *n* (%)	10407(16.6)	8660(15.9)	1747(21.6)	<0.001
Education: Illiterates, *n* (%)	34808(55.6)	30434(55.8)	4374(54.2)	0.006
Elementary school, *n* (%)	18179(29.0)	16006(29.4)	2173(26.9)	<0.001
Middle school or higher, *n* (%)	9608(15.3)	8084(14.8)	1524(18.9)	<0.001
Current married, *n* (%)	22668(36.2)	20244(37.1)	2424(30.0)	<0.001
# of alive children, mean (SD)	3.24(1.92)	3.27(1.92)	3.08(1.94)	<0.001
Log of income per capita, mean (SD)	8.27(1.53)	8.23(1.52)	8.58(1.58)	<0.001
ADL disabled, *n*(%)	15922(25.4)	12847(23.6)	3075(38.1)	<0.001
Cognitive impairment, *n* (%)	12437(20.9)	10542(20.3)	1895(25.2)	<0.001

### Relative Humidity and Temperature Exposure

The average relative humidity and temperature are also summarized in Table 1. The average relative humidity (mean ± SD) in the past year was 71.77 ± 8.01 %. The average temperature in the past year was 16.30 ± 3.92°C. The average temperature during the cold months of the previous year was 8.86 ± 5.57°C. During the warm months of the past year, the average temperature was 23.64 ± 2.42°C. Over the past year, the average daily maximum temperature was 21.08 ± 3.62°C, while the average daily minimum temperature has been 12.63 ± 4.54°C.

### Associations of Air Relative Humidity and Temperature With Cataracts

The average relative humidity over the past year was negatively correlated with the prevalence of cataracts. In the past year, each 1% rise in average relative humidity was associated with a 1.4% decrease in cataracts (OR: 0.99; 95% CI: 0.98–0.99). In contrast, the average temperature in the past year was positively related to the risk of cataracts. There was a 4% increase in cataracts with each 1°C increase in average temperature over the past year (OR = 1.04, 95%CI: 1.03–1.05). Based on the subgroup analyses, we found that the associations were robust across subgroups of cataract risk factors, including sex, urban/rural residence, and level of educational attainment. However, a non-significant effect of average temperature was found for urban population (Table 2).

**Table 2:** Effects of air relative humidity and temperature on Cataracts among Chinese older adults aged 65–105 during 2002–2018: Odds Ratios (OR) from GEE models.

	**All samples**	**Male only**	**Female only**	**Urban**	**Rural**	**0 year schooling**	**1+ year schooling**
	**OR[95%CI]**	**OR[95%CI]**	**OR[95%CI]**	**OR[95%CI]**	**OR[95%CI]**	**OR[95%CI]**	**OR[95%CI]**
Average relative humidity in the past year	0.99[0.98,0.99]^***^	0.99[0.99,1.00]^**^	0.98[0.98,0.99]^***^	0.99[0.99,1.00]^***^	0.98[0.97,0.99]^***^	0.98[0.97,0.98]^***^	1.00[0.99,1.00]
Average temperature in the past year	1.04[1.03,1.05]^***^	1.03[1.02,1.05]^***^	1.04[1.03,1.06]^***^	1.01[0.99,1.02]	1.10[1.08,1.11]^***^	1.05[1.03,1.06]^***^	1.03[1.02,1.04]^***^
**Covariates**
Rural residence (Urban*)	1.53[1.45, 1.62]^***^	1.61[1.48, 1.76]^***^	1.47[1.37, 1.57]^***^	–	–	1.39[1.30, 1.50]^***^	1.76[1.62, 1.91]^***^
East provinces (Middle/West*)	1.14[1.08, 1.21]^***^	1.21[1.11, 1.33]^***^	1.10[1.03, 1.19]^***^	1.32[1.22, 1.42]^***^	0.99[0.91, 1.08]	0.99[0.91, 1.06]	1.41[1.29, 1.54]^***^
Male (Female*)	0.69[0.64, 0.73]^***^	–	–	0.69[0.64, 0.75]^***^	0.68[0.62, 0.75]^***^	0.71[0.65, 0.78]^***^	0.67[0.62, 0.73]^***^
Age groups (65~79 years old*)							
80~89 years old	1.49[1.38, 1.61]^***^	1.71[1.52, 1.91]^***^	1.32[1.19, 1.46]^***^	1.42[1.29, 1.57]^***^	1.59[1.40, 1.79]^***^	1.37[1.21, 1.54]^***^	1.56[1.41, 1.72]^***^
90~99 years old	1.56[1.43, 1.70]^***^	1.99[1.75, 2.26]^***^	1.27[1.13, 1.43]^***^	1.57[1.40, 1.75]^***^	1.52[1.33, 1.75]^***^	1.36[1.19, 1.54]^***^	1.76[1.56, 1.98]^***^
100+ years old	1.58[1.43, 1.75]^***^	2.23[1.87, 2.65]^***^	1.27[1.12, 1.45]^***^	1.50[1.32, 1.71]^***^	1.65[1.41, 1.93]^***^	1.38[1.20, 1.59]^***^	1.81[1.54, 2.13]^***^
Years of schooling (0 year*):							
1~6 years	1.22[1.13, 1.30]^***^	1.17[1.05, 1.30]^***^	1.24[1.13, 1.37]^***^	1.31[1.19, 1.43]^***^	1.08[0.97, 1.21]	–	0.82[0.76, 0.89]^***^
7+ years	1.53[1.41, 1.66]^***^	1.53[1.35, 1.72]^***^	1.50[1.33, 1.68]^***^	1.67[1.51, 1.85]^***^	1.11[0.95, 1.29]	–	–
Current married (Unmarried*)	0.98[0.91, 1.05]	1.08[0.98, 1.19]	0.89[0.80, 0.98]^**^	0.96[0.88, 1.05]	0.97[0.87, 1.08]	0.94[0.84, 1.05]	1.00[0.91, 1.10]
# of alive children	0.99[0.97, 1.00]^*^	0.99[0.96, 1.01]	0.98[0.96, 1.00]^*^	0.98[0.96, 1.00]^**^	1.00[0.98, 1.02]	0.99[0.97, 1.01]	0.99[0.97, 1.01]
Log of income per capita	1.10[1.07, 1.12]^***^	1.13[1.08, 1.17]^***^	1.08[1.05, 1.11]^***^	1.16[1.12, 1.20]^***^	1.02[0.99, 1.05]	1.08[1.05, 1.11]^***^	1.11[1.08, 1.15]^***^
ADL disabled (Active*)	1.64[1.54, 1.74]^***^	1.56[1.40, 1.73]^***^	1.69[1.56, 1.83]^***^	1.73[1.60, 1.88]^***^	1.51[1.36, 1.67]^***^	1.69[1.56, 1.83]^***^	1.57[1.42, 1.74]^***^
Cognitive impairment (Active*)	0.96[0.89, 1.02]	0.99[0.88, 1.11]	0.95[0.87, 1.03]	0.96[0.88, 1.05]	0.96[0.87, 1.07]	0.97[0.89, 1.05]	0.95[0.85, 1.06]
Wave (2002*):
2005	1.03[0.95, 1.11]	0.95[0.84, 1.08]	1.08[0.98, 1.20]	0.96[0.87, 1.07]	1.11[0.98, 1.25]	1.01[0.92, 1.12]	1.06[0.94, 1.20]
2008	0.89[0.82, 0.97]^***^	0.80[0.70, 0.92]^***^	0.96[0.86, 1.06]	0.87[0.78, 0.97]^***^	0.92[0.81, 1.04]	0.86[0.78, 0.96]^***^	0.95[0.83, 1.08]
2011	0.94[0.85, 1.03]	0.84[0.72, 0.98]^**^	1.00[0.89, 1.13]	0.99[0.87, 1.12]	0.91[0.78, 1.05]	0.88[0.77, 0.99]^**^	1.04[0.90, 1.19]
2014	1.02[0.92, 1.12]	1.03[0.88, 1.21]	1.00[0.87, 1.14]	1.07[0.93, 1.23]	0.98[0.84, 1.15]	0.89[0.77, 1.02]^*^	1.21[1.04, 1.41]^**^
2018	1.00[0.92, 1.09]	0.94[0.82, 1.08]	1.03[0.92, 1.15]	0.98[0.88, 1.10]	1.10[0.95, 1.26]	0.92[0.81, 1.03]	1.12[0.99, 1.27]^*^

* *p <0.10*,

** *p <0.05*,

*** *p <0.01. OR, Odds Ratio; CI, confidence interval. Generalized estimation equation (GEE) were used. Dependent variables in all the 7 models are “Having Cataracts or not (Yes = 1, No = 0)”*.

We did sensitivity analyses to check the robustness of our findings *via* following analyses: using the average temperature in the warm months (Model I), the average temperature in the cold months (Model II), the average daily maximum temperature (Model III), and the average daily minimum temperature (Model IV). The results were consistent with our main findings (Table 3).

**Table 3:** Effects of air relative humidity and temperature on Cataracts: Odds Ratios (OR) from hierarchical GEE models.

	**Model I**	**Model II**	**Model III**	**Model IV**
	**OR[95%CI]**	**OR[95%CI]**	**OR[95%CI]**	**OR[95%CI]**
Average relative humidity in the past year	0.99[0.98, 0.99]^***^	0.99[0.98, 0.99]^***^	0.99[0.98, 0.99]^***^	0.99[0.98, 0.99]^***^
Average temperature in the cold months	1.03[1.02, 1.04]^***^			
Average temperature in the warm months		1.06[1.04, 1.07]^***^		
Average of daily maximum temperature			1.04[1.03, 1.05]^***^	
Average of daily minimum temperature				1.03[1.02, 1.04]^***^
**Covariates**	√	√	√	√

*p <0.10.

**p <0.05.

*** *p <0.01. OR, Odds Ratio; CI, confidence interval. Generalized estimation equation (GEE) were used. Dependent variables in all the 4 models are “Having Cataracts or not (Yes = 1, No = 0)”. All samples are included. Covariates are the same as in Table 2, the Odds ratios of which are not listed*.

**Figure 2:**
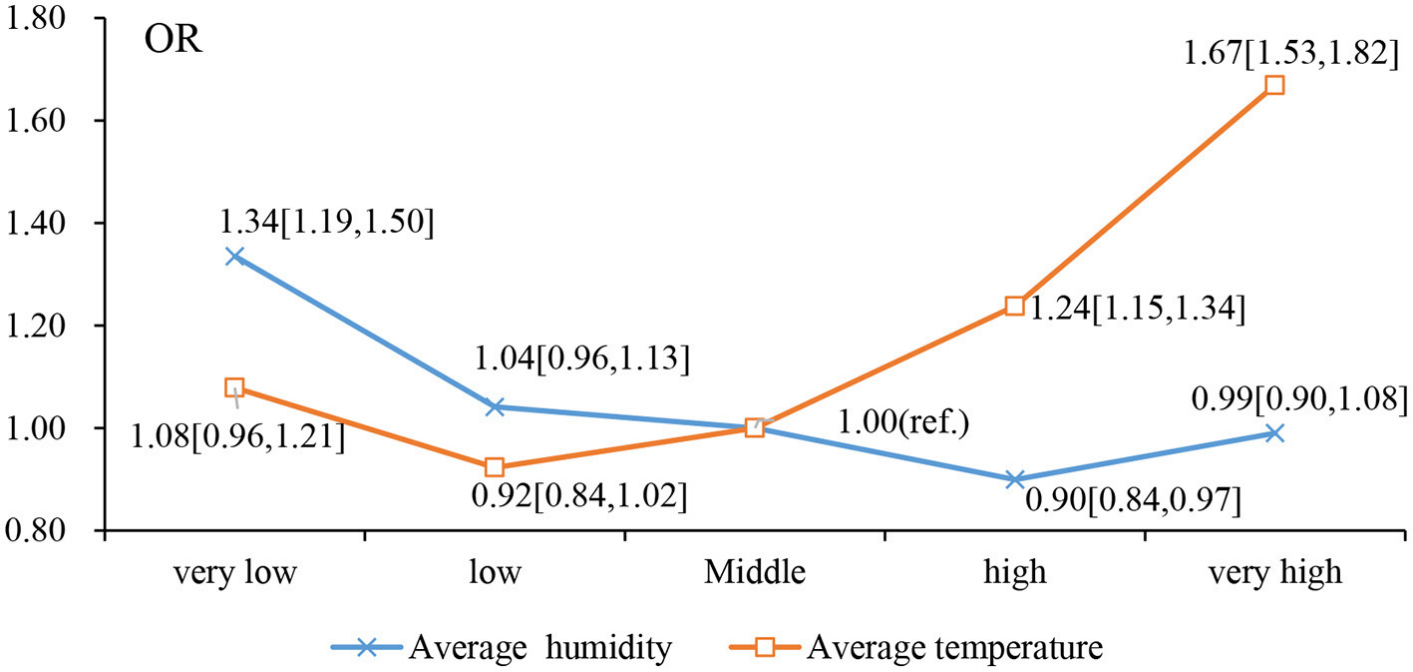
Effects of Categorical air relative humidity and temperature on Cataracts: Odds Ratios (OR) from Model I in Table 4.

In addition, in order to examine the potential nonlinear relationship of air relative humidity and temperature with cataracts, relative humidity and temperature were modeled as categorical variables with “middle” as a reference group. According to Table 4 and Figure 2, we observed a nonlinear J-curve relationship between the temperature and cataract prevalence. This means that while the temperature is generally positively associated with the prevalence of cataracts, particularly in the “low, middle, high, very high” interval, there is no noticeable trend when the temperature is in the “very low, low, middle” interval.

**Table 4:** Effects of Categorical air relative humidity and temperature on Cataracts: Odds Ratios (OR) from hierarchical GEE models.

	**Model I**	**Model II**	**Model III**
	**OR[95%CI]**	**OR[95%CI]**	**OR[95%CI]**
Average relative humidity in the past year			
Very low: <60	1.34[1.19, 1.50]^***^	1.37[1.22, 1.53]^***^	1.30[1.18, 1.44]^***^
Low: 60–69.99	1.04[0.96, 1.13]	1.10[1.01, 1.20]^**^	1.02[0.94, 1.10]
Middle: 70–74.99 (ref.)	1.00	1.00	1.00
High: 75–79.99	0.90[0.84, 0.97]^***^	0.86[0.80, 0.93]^***^	0.92[0.86, 0.99]^**^
Very high: ≥80	0.99[0.90, 1.08]	0.94[0.86, 1.03]	1.06[0.97, 1.16]
Average temperature in the past year			
Very low: <12°C	1.08[0.96, 1.21]		
Low: 12–14.99°C	0.92[0.84, 1.02]		
Middle: 15–16.99°C (ref.)	1.00		
High: 17–19.99°C	1.24[1.15, 1.34]^***^		
Very high: ≥20°C	1.67[1.53, 1.82]^***^		
Average temperature in the cold months			
Very low: <5°C		0.89[0.80, 0.99]^**^	
Low: 5–7.99°C		0.71[0.64, 0.78]^***^	
Middle: 8–9.99°C (ref.)		1.00	
High: 10–12.99°C		1.18[1.08, 1.28]^***^	
Very high: ≥13°C		1.34[1.23, 1.46]^***^	
Average temperature in the warm months			
Very low: <21°C			1.04[0.94, 1.15]
Low: 21–22.99°C			0.88[0.82, 0.96]^***^
Middle: 23–24.49°C (ref.)			1.00
High: 24.5–25.99°C			1.18[1.09, 1.27]^***^
Very high: ≥26°C			1.52[1.40, 1.65]^***^
**Covariates**	√	√	√

* *p <0.10*.

** *p <0.05*.

*** *p <0.01. OR, Odds Ratio; CI, confidence interval. Generalized estimation equation (GEE) were used. Dependent variables in all the 4 models are “Having Cataracts or not (Yes = 1, No = 0)”. All samples are included. Covariates are the same as in Table 2, the Odds ratios of which are not listed*.

## Discussion

We found significant associations of annual average relative humidity and temperature with the prevalence of cataracts. The odds ratios were 0.99 (95% CI: 0.98, 0.99) and 1.04 (95% CI: 1.03, 1.05) for annual average relative humidity and temperature, respectively. Furthermore, we found a nonlinear J-shaped relationship between temperature and cataracts prevalence. The associations indicated that extreme heat and low humidity may be linked to a higher incidence of cataracts in older individuals.

We found that lower humidity and higher temperatures were associated with an increased risk of cataracts. Although limited epidemiological studies have explored the health effects of humidity, similar findings have been reported in previous studies on other eye diseases. Based on a study of 100,636 participants, relative humidity was negatively related to allergic conjunctivitis (10). In Zhong et al.'s study (13), there is also a negative link between relative humidity and dry eye disease, supporting the evidence from a Korean study (11), suggesting that the moisture in the air might have contributed to the maintenance of the tear film on the ocular surface. In support of our findings, a recent study indicated that exposure to low relative humidity on tear film adversely affected the rate of evaporation, the thickness, and stability of the lipid layer, and the production of tears; this resulted in significant postoperative discomfort. A dry environment increases light scattering, especially in older adults, who need to blink more frequently to prevent their corneas from becoming dehydrated (23).

Regarding temperature, Miranda et al. reported that senile cataract develops earlier and is more prevalent in warm regions, and that cataract prevalence increases with increasing temperature (24). According to Chatterjee et al., similar results were observed in Punjab, India (25). According to another study, plains tend to have higher average annual temperatures and a higher prevalence of cataracts than mountainous areas (26). In a study by Kodera et al., they computed the change in lens temperature as a result of exposure to ambient conditions in people 50 to 60 years of age living in tropical and temperate regions. It was observed that a strong correlation existed between the prevalence of nuclear cataracts and the computed cumulative thermal dose in the lens (27).

Low humidity may affect cataract prevalence by drying out the airways, resulting in hyperosmolarity, which stimulates nerves to produce reflex responses and may release inflammatory biomarkers (28). It has been proposed that inflammatory cytokines and growth factors in tears are altered by exposure to dry environments, thereby interfering with the immune response's homeostasis (29–31). Through the cornea, the corneal surface is directly influenced by the exterior temperature of the eye. As blood transmits body temperature to the eye, high ambient temperatures may result in thermal damage to ocular structures (32). Lifelong exposure to small increases in temperature may, therefore, contribute to the accelerated aging process of the lens by accelerating the metabolic rate of the lenticular epithelium (33). Nandi demonstrated that the transient and subtle temperature elevations in the lens of the eye could result in protein cross-linking through AGEs and cause age-related cataracts (34). Studies in animals have shown that cataracts may develop when the temperature of the eye's lens increases with the temperature of its surroundings. Brown Norway rats exposed to 35 ± 2°C for 3 weeks developed cataracts at a higher rate than those exposed to 24 ± 2°C (35). Furthermore, organ cultured rat lenses incubated at 40–50°C developed cortical cataracts (36). Evidence generated from animal models is not directly comparable to that in humans. The evidence generated from animal models does not directly indicate that humans develop cataracts in high-temperature environments due to similar mechanisms of temperature rise, but the possibility remains (34).

This study contains several limitations. First, relative humidity and temperature are only measured from ambient data at the city level (not from indoors), since personal exposure varies with the ventilation in a house and the movement of people inside the house. Second, except for humidity and temperature, we were unable to assess the possible confounding effects or interactive effects of other environmental exposures (e.g., PM_2.5_ and NO_2_) on cataracts. Furthermore, further research from the perspective of molecular biology is needed to investigate the causal relationship between relative humidity and temperature and cataract development.

To our knowledge, this is the first study in China to investigate the relationship of relative humidity and temperature with cataracts in older adults. Due to the drastic climate change and population aging across the globe, it is intriguing to study the relationship between temperature, relative humidity and age-related conditions. We believe that our finding will promote the attention of relevant personnel on eye health issues related to weather change to improve the situation of cataracts and other eye diseases among older adults in China and improve their quality of life.

In conclusion, we found that the average relative humidity in the past year was inversely associated with cataract likelihoods in older adults and a J-shaped positive association between temperature in the past year and cataract likelihoods in older adults. Our findings indicated that extreme heat and low humidity were independently associated with higher likelihoods of cataracts in older adults.

## Data Availability Statement

The raw data supporting the conclusions of this article will be made available by the authors, without undue reservation.

## Author Contributions

YY and HC designed the study. HC performed the analyses. XL and XG drafted the paper. KH, YZ, YY, and HC reviewed the paper. All authors contributed to the article and approved the submitted version.

## Funding

This work was supported by National Natural Science Foundation of China (42001013, 81561128020, and 81872920) and Natural Science Foundation of Hunan China (2020JJ4087), and the State Laboratory of Sub-tropical Architecture of China (2020ZB10).

## Conflict of Interest

The authors declare that the research was conducted in the absence of any commercial or financial relationships that could be construed as a potential conflict of interest.

## Publisher's Note

All claims expressed in this article are solely those of the authors and do not necessarily represent those of their affiliated organizations, or those of the publisher, the editors and the reviewers. Any product that may be evaluated in this article, or claim that may be made by its manufacturer, is not guaranteed or endorsed by the publisher.
